# The Strategic Advisory Group of Experts (SAGE) on Immunization—Past, Present and Future

**DOI:** 10.3390/vaccines12121402

**Published:** 2024-12-12

**Authors:** Melanie Marti, Hanna Nohynek, Philippe Duclos, Katherine L. O’Brien, Joachim Hombach

**Affiliations:** 1 Department of Immunization, Vaccines and Biologicals, World Health Organization, CH-1211 Geneva, Switzerland; 2 Finnish Institute for Health and Welfare, 00271 Helsinki, Finland; 3 Formerly Department of Immunization, Vaccines and Biologicals, World Health Organization, CH-1211 Geneva, Switzerland

**Keywords:** Strategic Advisory Group of Experts (SAGE) on Immunization, SAGE, World Health Organization advisory group, global immunization policy, immunization strategy, vaccination policy, vaccination recommendations

## Abstract

Background/Objectives: In November 1999, WHO established the Strategic Advisory Group of Experts (SAGE) on Immunization as a multidisciplinary group of experts to provide high-level recommendations on vaccines and immunization. Methods: This review provides an overview of SAGE’s work in the past 25 years. It further outlines the processes and methods currently used by SAGE and highlights some of its major achievements. Results: SAGE’s global policies have driven action toward eradication, elimination and disease control and addressed the optimization of vaccination and immunization. In total, 27 major policy positions on vaccines/vaccine-preventable diseases have guided global public health. During times of epidemics and pandemics, interim recommendations issued by SAGE have responded iteratively in real-time to provide evidence-driven response policies. SAGE is an adaptive advisory group that has modified its procedures and working approaches to meet the evolving challenges in public health and stay up-to-date with evolving scientific and guideline development standards. Conclusions: Over the last quarter century, SAGE has significantly contributed to shaping the immunization landscape. It has achieved and maintained a high level of integrity and credibility. The advisory group continues to be an authority in global public health, and its recommendations have profound implications for the health of individuals and populations across the globe.

## 1. Introduction

The World Health Organization (WHO) was established with the objective of the attainment of the highest possible level of health for all people. In its constitution, the organization’s function was outlined “to act as the directing and co-ordinating authority on international health work” and to “make recommendations with respect to international health matters [1]”. Consequently, WHO provides leadership, including technical and normative guidance, on the control, elimination and eradication of vaccine-preventable diseases and the immunization programs to achieve those goals.

Vaccines are one of the most successful public health interventions of all time [2]. Recent modeling sought to quantify the public health impact of vaccinations globally from the inception of the Expanded Programme on Immunization 50 years ago [3]. Since 1974, the year the World Health Assembly requested WHO to provide technical advice and assistance to countries on the use of vaccines and recommended that Member States develop or maintain immunization and surveillance programs [4], vaccination against 14 diseases has averted over 154 million deaths, including 146 million among children younger than five years, of whom 101 million were infants younger than one year. During this period, vaccination has accounted for 40% of the observed decline in global infant mortality, 52% in the African region. In 2024, a vaccinated child younger than 10 years is 40% more likely to survive to their next birthday relative to a hypothetical scenario of no vaccination [3].

Many resources are devoted to the development and testing of vaccines, leading, ultimately, to their licensure and use in populations. Nevertheless, market availability of the products alone neither ensures their availability to all in need nor their appropriate use. Additional mechanisms are required to provide vaccines equitably to individuals worldwide who need them. This encompasses not only funding mechanisms to assure fair distribution and access but also strategies and policies developed to guide vaccination programs and to ensure the highest impact of vaccine utilization.

WHO established the Strategic Advisory Group of Experts (SAGE) on Immunization as a multidisciplinary group of independent experts to provide high-level guidance to WHO on vaccines and immunization. SAGE takes a population health approach with a mandate of issuing advice on vaccines of major public health importance, spanning across the life course, for programmatic use, including during health emergencies. While SAGE aims to issue global guidance targeted at regions and countries across all income strata (high-, middle- and low-income countries), its impact may be highest in low- and middle-income countries.

While SAGE is charged with the provision of guidance to WHO, SAGE also assumes, through WHO, advisory functions, inter alia, for Gavi, the Vaccine Alliance and the Global Polio Eradication Initiative (GPEI). In that function, SAGE’s recommendations are also highly consequential for financial investments by technical and other partners, manufacturers and funders.

## 2. History of SAGE

First established in 1999 through the merging of two WHO previous advisory groups [5,6] (The Scientific Advisory Group of Experts (SAGE) of the WHO Program for Vaccine Development) and the Global Advisory Group (GAG) of the Expanded Program on Immunization.), SAGE was initially set up to advise and report to the director of the former WHO Department of Vaccines and Biologicals—the predecessor of the Department of Immunization, Vaccines and Biologicals—on the strategic objectives of WHO’s immunization program.

The Global Immunization Vision and Strategy (GIVS) 2006–2015 [7], launched in 2005, was the first-ever multi-year partnership framework for realizing pre-defined immunization-related goals grouped in four strategic areas (Strategic areas: First, protecting more people in a changing world; Second, introducing new vaccines and technologies; Third, integrating immunization, other linked health interventions and surveillance in the health systems context; Fourth, immunizing in the context of global interdependence.). In parallel, SAGE underwent an adjustment of its scope and terms of reference and operating processes to ensure alignment with the guiding principles of the GIVS, outlining its tasks pertaining to the GIVS and enabling it to formulate global immunization strategies and policies. SAGE was, therefore, mandated to provide guidance on immunization priorities, advise on matters pertaining to vaccine development and research, issue immunization policies and advise on strategies extending beyond childhood immunization to all major vaccine-preventable diseases throughout all age groups. SAGE, therefore, evolved ”into a body overarching global immunization” [8,9]. SAGE was asked to advise the WHO Director-General on the adequacy of progress towards the achievement of the GIVS objectives and to identify major issues and challenges.

SAGE’s role was again updated in 2011 when the Global Vaccine Action Plan (GVAP) was established as the strategic framework for the new decade [10]. SAGE was asked to determine the ambitions for 2011–2020 and took on a critical role in the GVAP oversight. SAGE provided annual reviews of progress facilitated by its Decade of Vaccine Working Group. SAGE’s assessment of progress on the GVAP goals fed into a subsequent review and assessment by ministers of health through WHO’s Executive Board and the World Health Assembly.

The co-creation process of the current decade’s vaccine and immunization strategy, Immunization Agenda 2030 (IA2030) [11,12], included a broad range of national and international stakeholders that helped shape the vision and strategy for 2021–2030 [13]. IA2030 aspires to ensure that the vision, strategic priorities and goals are strongly driven by and aligned with country needs. Once a year, SAGE provides an independent review and guidance across the IA2030 strategic priority areas, informed by a detailed analysis of annual progress as laid out by the monitoring and evaluation framework.

Notwithstanding the impact of the above-mentioned global immunization strategies on the scope of SAGE, two external evaluations have also shaped SAGE’s operations. These evaluations improved the advisory group’s functioning and helped to more strongly root it within the global, regional and national immunization decision-making context.

A first review looking at the overall immunization advisory architecture of WHO was conducted in 2007. It confirmed SAGE as the principal advisory committee, which makes recommendations on all aspects pertaining to vaccine and immunization policies and strategies. The evaluation stressed, though, the importance of stronger linkages between SAGE and the Regional Immunization Technical Advisory Groups (RITAGs). Further evaluations of the impact and functioning of SAGE and of WHO’s immunization advisory committees took place over the years.

In 2018, the last independent external evaluation of SAGE coincided with the launch of WHO’s 13th General Programme of Work as well as with the planning process for the 2021–2030 global immunization strategy [14]. It also built on a corporate evaluation of WHO’s normative function, initiated in 2017 [15], and emphasized the importance of serving the needs of countries. Overall, the evaluation concluded that the immunization stakeholder community considered SAGE extremely valuable and well respected, played a critical and leadership role in global immunization and was strong in providing evidence-based recommendations. The overall SAGE modus operandi with the consensus-based decision-making, the use of Working Groups, and the robust scientific advisory structure using evidence-based methodologies were considered highly effective. At the same time, several areas for improvement were highlighted. In response to these, the SAGE terms of reference [16] were revised to more clearly reflect the primary goals and scope of SAGE. A more systematic and transparent agenda-setting process was implemented, and the interactions between SAGE, RITAGs and National Immunization Technical Advisory Groups (NITAGs) were strengthened further. Moreover, additional guidance on what constitutes a conflict of interest for SAGE members and those providing input to the SAGE reviews of evidence was put in place.

## 3. SAGE’s Regular Proceedings

As of today, SAGE’s main purpose is to provide normative and strategic guidance on the use of existing or novel vaccines within national immunization programs. SAGE is advisory to the Director General of WHO, and all major vaccine- and immunization-related topics for which WHO requires strategic and policy advice are reviewed by SAGE. For its work, SAGE also relies on technical input from other more specialized advisory groups (see below).

SAGE is engaged in communication with regions and countries via RITAGs and NITAGs. Interactions with NITAGs are facilitated through the Global NITAG Network (GNN) [17] and regional NITAG networks. RITAGs are concerned with regional immunization policies and play an important role in the adaptation of global recommendations issued by SAGE into the regional- and country-level guidance. For example, in response to SAGE’s 2020 recommendation to prioritize the uses of COVID-19 vaccines in the context of limited supply, the European Technical Advisory Group of Experts on Immunization (ETAGE) adapted those recommendations based on regional epidemiology, available literature, vaccine impact modeling and published preliminary recommendations of selected NITAGs in the region [18]. In return, national-level requests for strategic and policy advice and country experiences are considered in SAGE’s work.

SAGE is composed of 15 internationally renowned experts with a strong devotion to public health, acting in their individual capacities. Candidatures are solicited through a public call for nominations. Experts are selected across a wide spectrum of academic disciplines, specialty areas and professions. Above all, SAGE members are selected based on their scientific excellence, professional experience, and seniority while ensuring diversity not only through the vast range of expertise represented on SAGE but also by appointing a geographical and gender-balanced advisory group. Members of SAGE are not remunerated for serving on SAGE and should be free of vested interests. Therefore, upon appointment and ahead of each meeting, SAGE members are asked to declare potential interests that are then assessed and managed by the WHO Secretariat and disclosed publicly ahead of each meeting [19].

SAGE meets face-to-face biannually, generally for 3 to 4 days. Meetings are scheduled years in advance and are structured into open and closed sessions. Plenary sessions, widely attended by immunization stakeholders and Members State representatives, are convened for the purpose of exchanging information and discussing policy options. Representatives of different stakeholder groups, including UN partner agencies, governmental and non-governmental organizations, funders and manufacturers via umbrella organizations, can attend the open session upon invitation, which, since the COVID pandemic, has been organized in a hybrid mode of both in-person and online. To ensure transparency and wide engagement of the immunization community, policy recommendations are made in the open session. The specific wording of the recommendations may be refined in the closed SAGE sessions, which also serve for technical briefings and managerial issues.

On special occasions, as at the onset or during public health emergencies such as Ebola and COVID-19, SAGE is convened as necessary for extraordinary sessions in order to assure that policy guidance is provided in a real-time manner commensurate as required by the public health emergency and as the evidence allows.

**Figure 1:**
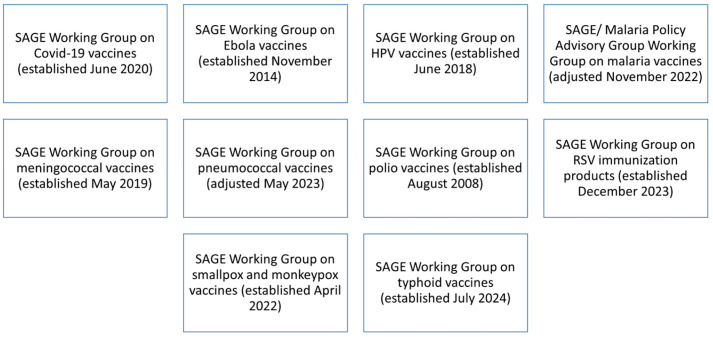
Current SAGE Working Groups (November 2024).

Topic-specific SAGE Working Groups are set up to collate and synthesize evidence and policy options for consideration by the full SAGE committee. Working Groups are established following an open call for membership and are usually time-limited. Two SAGE members ensure that liaison between SAGE and the respective Working Group [20] (see Figure 1).

Following each meeting, SAGE presents its policy recommendations to WHO’s Director General for formal endorsement. Only thereafter can SAGE’s advice be considered as official WHO guidance, most often reflected in WHO vaccine position papers. WHO publishes these vaccine position papers to provide global vaccine and immunization recommendations for diseases that have a major public health impact at a global or regional level. Vaccine position papers can be considered the flagship of global immunization guidance and are subject to a rigorous development process, which entails extensive peer-review by staff across the different levels of the organization as well as by external subject-matter experts. Over the course of the past 25 years, a total of 78 new or updated vaccine position papers (or closely related guidance) have been published on a total of 27 vaccines/vaccine-preventable diseases and two cross-cutting immunization-related topics (behavioral and social drivers of vaccine uptake and pain mitigation at the time of vaccination) (Table 1).

SAGE issues policy recommendations on vaccines against diseases of major public health importance. Policy recommendations are normally directed at a product class and indication, and product-specific considerations are only mentioned if they are public-health-relevant. Vaccines will be considered for policy if the manufacturer is regulated by a national authority of high competency (WHO-listed authority, previously termed maturity level 3 or higher). Besides the policy recommendations, WHO has a program for the prequalification of vaccines for public procurement [21]. This process is independent of the recommendation process and looks primarily at the quality, safety and efficacy of vaccines, as well as their programmatic suitability. In contrast to policy recommendations, WHO prequalification is product-specific.

Evidence-based decision-making in public health emphasizes that decisions should be informed by the best available scientific evidence as well as other criteria, such as context, equity, feasibility of implementation, affordability, sustainability and acceptability to stakeholders [22,23]. SAGE requests that all evidence on which its guidance is based be publicly available. SAGE has always recognized the necessity of providing guidance based on recent, ideally high-certainty data, assessed by internationally recognized methods, approaches and best practices. The processes, methods and approaches used by SAGE to develop evidence-based recommendations have been published as an authoritative document [24] and follow WHO’s overall guidance for its core normative work.

The Grading of Recommendations, Assessment, Development and Evaluation (GRADE) approach [25] is an approach to rate the certainty of the best available evidence in developing healthcare recommendations. GRADE has been adopted by WHO and many other national and international organizations, with SAGE being an early adopter, having applied this methodology since April 2007 [26].

GRADE feeds into the Evidence-to-Decision table [27], a decision-support framework that encourages an explicit accounting of the panel’s judgments based on the available evidence. These frameworks encourage the panel to consider the benefits and potential harms of an intervention but also other criteria, including resource requirements, cost-effectiveness, acceptability, feasibility and values and preferences. Documenting the assessment of each of these criteria in a systematized and transparent way allows interested parties to comprehend the thought process behind the recommendation. It further enables regional and country immunization programs to better adapt guidance to their local context.

In the context of immunization policies, SAGE follows the GRADE approach and may provide countries with strong or conditional recommendations [28]. Strong recommendations may be issued when the evidence base has been judged to be moderate to high. Strong recommendations may also be issued exceptionally based on low or very low certainty of evidence if the intervention reduces mortality in life-threatening situations or if adverse events following the intervention are deemed to be acceptable [28].

Conditional recommendations may be issued when the evidence-base has been judged to be low to very low, or when there is a close balance between the desirable and the undesirable consequences of an intervention.

All SAGE recommendations may be restrictive to a certain condition, setting, geography or population.

SAGE may issue off-label recommendations. Off-label recommendations for public health use [29] differ from the use indication on the product label provided by the manufacturer and reviewed by regulatory authorities in case there is a significant benefit from it to the target populations. The importance of SAGE’s off-label recommendations is twofold: the recommendations provide global guidance to NITAGs considering going beyond the use-indication, be it in emergency situations or to optimize the efficiency and reach of the intervention at programme level. In addition, these recommendations provide a signal to manufacturers to consider seeking label extensions by national regulatory authorities. Off-label recommendations span from use in target (e.g., pregnancy) or age groups beyond those of the label, fractional doses or different routes of administration and, most commonly, alternative (often reduced) dosing schedules. Off-label recommendations are based on evidence and are the result of a benefit–risk assessment.

One highly consequential off-label recommendation resulting in great public health benefit was issued by SAGE in 2022 [30] when it was concluded that a single-dose of Human Papillomavirus (HPV) vaccine delivers comparable protection against cervical cancer as the 2-dose schedule in girls aged 9–14 years. This recommendation became a game-changer for vaccination programs, addressing serious supply constraints, easing programmatic introduction and cutting costs, thereby enabling countries to reach more girls with this life-saving vaccine. Since this recommendation, several manufacturers have initiated studies on single-dose schedules with the aim of seeking a product label variation.

**Figure 2:**
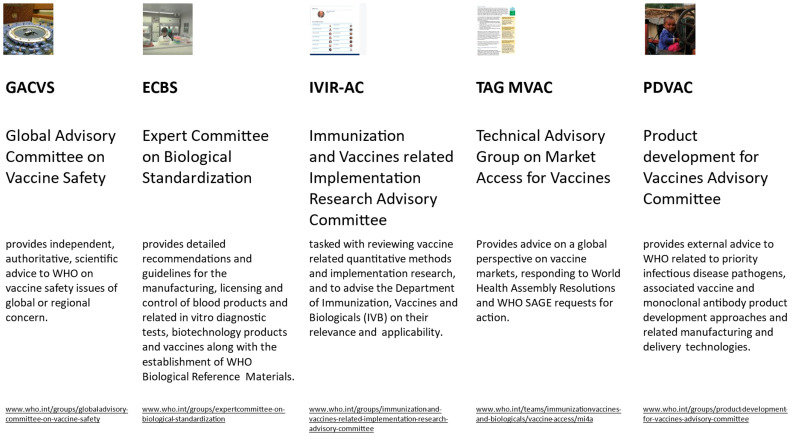
Other WHO advisory groups and committees relevant to immunization.

The work of complementary WHO immunization-related advisory committees, with specialized remits, provides a unique resource for SAGE and plays an important role in the development of policy recommendations (Figure 2).

Three advisory groups are of particular relevance to SAGE:

The Product Development for Vaccines Advisory Committee (PDVAC) [31] is an advisory group of experts that provides advice to WHO on research matters related to priority infectious disease pathogens and vaccines yet under development. Early engagements with SAGE help to define key data needs for future policy development at a time when some of the questions can be integrated into late-stage vaccine development. As a result, relevant and timely data may inform SAGE policy recommendations once candidate vaccines mature to obtain regulatory approval. The Global Advisory Committee on Vaccine Safety (GACVS) [32] is an advisory group dedicated to vaccine safety, in particular, to conduct post-introduction safety assessments. In special situations, SAGE may request a GACVS review on prelicensure vaccine safety data. The risk assessment by GACVS, as well as advice on pharmacovigilance, will be considered in SAGE’s benefit–risk assessment and formulation of policy.

The Immunization- and Vaccine-related Implementation Research Advisory Committee (IVIR-AC) [33] provides an independent appraisal of and advice on questions related to vaccine implementation and, in particular, an appraisal of methods used for the quantitative effects of vaccines, such as the population impact and cost-effectiveness analysis, which are important data to shape SAGE’s recommendations on policy and vaccination strategies. IVIR-ACs emphasize the suitability of methods, while the outputs of the quantitative analysis will be scrutinized by SAGE and its Working Groups.

## 4. SAGE Recommendations in Special and Emergency and Humanitarian Situations

SAGE’s objective is to develop timely recommendations with an emphasis on equity and optimal population protection. In emergency situations, recommendations need to be issued, often on the basis of less comprehensive information, which may take the form of interim recommendations. Managing uncertainty in issuing policy recommendations requires well-balanced recommendations, maximal transparency regarding prevailing data gaps and the effective mitigation strategies of supply constraints. In all these circumstances, SAGE may issue off-label recommendations after careful assessment of the risks and benefits. In emergency situations, SAGE also advises on the use of emergency-use-authorized (or WHO emergency use listing [34]) vaccines and, in exceptional circumstances, on expanded access strategies on investigational products.

### 4.1. Decision-Making in the Context of Uncertainty

SAGE is often confronted with the task of issuing policy recommendations, while data generated during the clinical trial program used for registration are comprehensive but may be limited in terms of enrolled study populations, sample size, duration of follow-up or other elements desirable for robust policy recommendations. While this situation occurs inevitably in the context of emergency situations (see below), it also often applies to new vaccines registered for routine use. In any of these circumstances, SAGE will request additional research and more comprehensive data and adapt/complete its recommendations if needed. Without discussing the examples in detail, reference can be made to SAGE’s decision-making in the context of uncertainty when advising on dengue and malaria vaccination as two examples.

#### 4.1.1. Dengue Vaccination

In 2016, WHO issued the first WHO position paper on dengue vaccination, based on a comprehensive review of data generated from a multicentric clinical trial on a first-in-class product. Noting residual data gaps regarding the safety of the vaccine, WHO issued a conditional recommendation for limiting vaccine use in geographic areas with a high burden of disease. This was a precautionary measure against an, at that time, unproven risk of enhanced disease in vaccine recipients not previously exposed to dengue prior to vaccination. This recommendation was backed by an extensive benefit–risk analysis. Following cases of severe disease in several dengue-vaccinated children in a school-based vaccination program, a major controversy arose. While it was not possible to assess if cases occurred as a result of enhanced disease following vaccination or were cases of breakthrough disease, at WHO’s request, the manufacturer conducted an additional analysis of clinical trial samples with a newly developed diagnostic procedure. SAGE rapidly reconvened in November 2017 to assess the emerging evidence from a retrospective analysis of clinical trial data. The data confirmed the previously hypothetical excess risk of severe dengue in seronegative vaccine recipients compared to seronegative non-vaccinated individuals while confirming long-term protection in seropositive individuals. While the initial positive benefit–risk assessment did not change, SAGE provided revised recommendations in April 2018 that were reflected in an updated vaccine position paper [35], providing the option of serologic pretesting of individuals prior to vaccination.

#### 4.1.2. Malaria Vaccination

In 2015, SAGE and the Malaria Policy Advisory Committee (MPAC) (For the malaria session, SAGE was joined by the Malaria Policy Advisory Committee (MPAC) and the conclusions and recommendations concerning the malaria vaccine were those of both committees. MPAC is now called the Malaria Policy Advisory Group (MPAG). MPAG provides independent, strategic advice to WHO on all technical areas relating to malaria control and elimination.) in a joint session, assessed the evidence of a first-generation malaria vaccine, which had received a positive regulatory opinion (Using the EU-Medicines for all procedure (EU-M4all) to provide an opinion on medicines and vaccines for human use intended exclusively for markets outside of the European Union (Article 58 of the EMA Regulation (EC) No 726/2004).) by the European Medicines Agency. While the benefit–risk assessment was positive, potential safety signals had emerged from a detailed review of the phase three clinical trials program (i.e., meningitis, cerebral malaria and excess of deaths from all causes in girls compared to boys). In addition, there was uncertainty about the feasibility of implementing the vaccination schedule in a routine immunization program because it required new touchpoints. To address those uncertainties and ancillary research questions, it was recommended that additional evidence be collected through a staged pilot implementation in several countries, such as the malaria vaccine implementation program (MVIP). A framework for policy decision-making was developed for MVIP, which prospectively defined the data required for a more generalized policy recommendation. When those data became available, a second joint SAGE/MPAG^3^ evidence review session was convened in October 2021, resulting in a recommendation and WHO position paper in March 2022 [36]. RTS,S was prequalified in July 2022, which then allowed for a malaria vaccine roll-out (with Gavi support) throughout *P. falciparum* malaria-endemic countries.

### 4.2. SAGE in the Context of Outbreaks and Pandemics

SAGE also serves as WHO’s vaccine and immunization advisory body for the use of vaccines in the context of public health emergencies. Urgent strategic and policy advice by SAGE was required in the context of the H1N1 pandemic in 2009, the yellow fever outbreak in 2016, the Ebola outbreaks in 2013–2016 and 2018–2020, the COVID-19 pandemic in 2020–2023, and more recently, the mpox outbreaks in 2022–2023 and 2024, covering six of the seven Public Health Emergencies of International Concern (PHEIC) declared by WHO. Only the Zika virus PHEIC did not have a vaccination component, but the process triggered the development activities of this much-needed vaccine.

SAGE’s engagement within these outbreaks varied, as described below. In each case, the aim has been to provide timely, evidence-based, pragmatic public health policy recommendations on vaccine use and prioritization in settings with incomplete and evolving evidence regarding the pathogen, epidemiology, risk factors for infection and disease and the vaccines.

### 4.3. Multi-Country Yellow Fever Outbreak

In 2016, large-scale yellow fever outbreaks were reported from central Africa, sharply increasing the demand for already limited yellow fever global vaccine supplies. WHO needed to swiftly respond to the rapidly depleting vaccine stockpile. In July 2016, WHO reviewed potential vaccine dose-sparing options, namely fractional doses using different modes of administration and manufacturers’ reports on potency and other criteria. Given the urgent need for WHO policy guidance to its Member States and to the wider vaccine community, SAGE was asked to appraise an assessment of evidence prepared by the secretariat, which was then released as WHO guidance. This was an exceptional process, given the lack of time for the formal SAGE review of evidence and deliberation processes that usually inform and result in its recommendations [37]. A formal SAGE assessment of the available evidence, as well as the country’s experience with implementing the fractional dose policy, was conducted subsequently by SAGE in October 2016. The advisory group recommended that a fractional dose could be used as part of an exceptional response [38], and these recommendations were reflected in an addendum to the WHO yellow fever position paper [39]. SAGE was also asked to provide strategic advice leading to the development of the Eliminate Yellow Fever Epidemics (EYE) strategy [40], a global effort to tackle, in a co-ordinated manner, the increased risk of yellow fever epidemics for the period 2017–2026. Since then, the research agenda advocated for by SAGE has largely been implemented, and the fractional dose strategy for yellow fever outbreaks has been recommended in other emergency situations.

### 4.4. The 2009 H1N1 Influenza a Pandemic

In the context of the 2009 Influenza A (H1N1) pandemic, an ad hoc Working Group on H1N1 vaccines was established. Their assessment, alongside regional perspectives, led SAGE to issue emergency recommendations on H1N1 vaccines during an extraordinary meeting on 7 July 2009. SAGE noted that “its recommendations reflected the pandemic’s current estimated severity and, as the situation evolves and more evidence becomes available, the recommendations may need to be revisited” [41]. SAGE noted with great concern that in the current situation, a small number of industrialized countries would have access to most of the global vaccine supply over the next 12 months through purchase agreements, limiting vaccine availability for the rest of the world and especially in developing countries. Therefore, SAGE emphasized the importance of striving to achieve equity among countries concerning access to the vaccines developed in response to the pandemic. While H1N1’s disease severity and pandemic trajectory, fortunately, did not necessitate an extended global vaccination response, the pandemic influenza preparedness (PIP) framework for the sharing of influenza viruses and access to vaccines and other benefits was developed and adopted by the Sixty-fourth World Health Assembly in 2011.

### 4.5. COVID-19 Pandemic

While the world was caught by surprise with the emergence of a previously unknown virus, SARS-CoV2, in late 2019 and its unprecedented spread starting in early 2020, the initial absence of vaccines provided SAGE with several months to prepare for their arrival. During this time, SAGE started proceedings under the assumption that vaccine development would be successful. In anticipation of the urgently awaited results of vaccine clinical trials, SAGE, supported by its COVID-19 Working Group set up in June 2020 [42] and several subgroups (prioritization, evidence and modeling subgroup), went beyond its usual guidance. SAGE initially developed an ethics- and value-based framework for countries on the prioritization of population groups for the anticipated initial limited vaccination supply [43].

As the COVID-19 Vaccines Global Access (COVAX) initiative, the vaccines pillar of the Access to COVID-19 Tools (ACT) Accelerator [44] was being set up—a global collaboration to accelerate the development, production and equitable access to COVID-19 tests, treatments and vaccines—and SAGE was naturally confirmed as its advisory body responsible for issuing evidence-based immunization policy recommendations.

Upon the arrival of the first vaccine doses, SAGE’s functioning was pressure-tested. The committee needed to act at speed while keeping abreast of the ever-evolving evidence, often not even published in the scientific literature. Informed by a living systematic review of the evidence [45] and close exchanges with the global research community, and informed by manufacturers, SAGE, in 2021, developed a noteworthy number of 23 product-specific interim recommendations and other related COVID-19 publications, such as guidance on extended COVID-19 immunization schedules for immunocompromized individuals.

**Figure 3:**
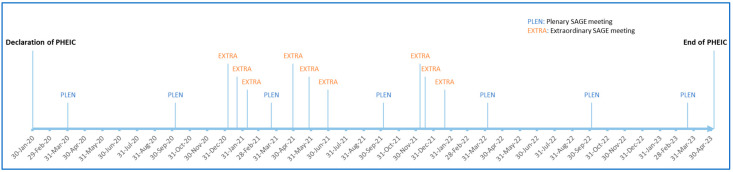
Occurrence of ordinary and extraordinary SAGE meetings in the period of the COVID-19 PHEIC.

The COVID-19 Working Group met weekly over the course of the pandemic, accounting for more than 100 meetings. SAGE convened a record number of 17 times following the declaration of the COVID-19 PHEIC in 2020 to its lifting in 2023 (Figure 3).

The COVID-19 pandemic put exceptional strain on SAGE, the SAGE COVID-19 vaccines Working Group and on WHO staff. Meeting the needs of Member States, the global public health community, COVAX and many other stakeholders was the top priority for WHO’s vaccine policy and strategy team and tremendously challenging given the evidence gaps, the need for real-time review and synthesis of newly available evidence and the soar in workload this demanded. For SAGE members, the surge in the number and scale of policy recommendations that needed to be formulated meant dedicating a huge amount of time to keep up with rapidly emerging data, revision of documents and attending multiple virtual meetings, in addition to the equally demanding surge in day-to-day activities of their regular employment.

Additionally, the necessity for SAGE to make decisions expeditiously in conditions of significant uncertainty, such as in the context of constant virus evolution and changing population immunity, was unprecedented. Unlike regular recommendations, where SAGE reviews data across a class of vaccines against a specific antigen, the COVID-19 pandemic forced SAGE to issue product-specific recommendations across the various vaccine platforms authorized by regulatory bodies using emergency procedures. This led to a massive increase in supplementary work, both for SAGE and its WHO secretariat.

The lessons learned during COVID-19 fed into updated guidance on the development of WHO vaccines and immunization policy and strategic guidance. Post-pandemic, SAGE methods and procedures were adjusted, and the type of guidance issued was more clearly defined into three categories: formal WHO recommendations issued following a comprehensive evidence review of disease epidemiology and vaccine performance compiled over an extended period; rapid advice guidance, issued within a week to three months; emergency guidance, developed within hours to days of an emergency notification, necessitating a potential vaccine response [24].

During the pandemic, many NITAGs relied on and adapted WHO’s recommendations to their respective national contexts. A 2021 survey of 44 countries confirmed that SAGE recommendations concerning COVID-19 vaccines were assessed to be comprehensive and timely. Furthermore, they were seen as easy to access, understand and adapt [46].

## 5. Looking Ahead and Conclusions

SAGE’s greatest strength emerges from those individuals serving on it. Over the years, passionate and committed experts in the field of vaccines and immunization have dedicated very significant time and energy to serve on and/or chair the panel and its Working Groups. Experts have made outstanding contributions and have genuinely committed to advancing the global immunization agenda and have left a lasting imprint. Continuing to attract the world’s most distinguished vaccine and immunization experts and ensuring their engagement remains the cornerstone of SAGE’s functioning.

The steadfastness of the advisory group comes from the adherence to the guiding principles of independence, transparency and scientific rigor. Mechanisms to ensure adherence to these principles have evolved over time but have always remained the key pillar of SAGE’s standing.

Nevertheless, the progressive realization of these principles remains an ongoing effort. The relationship between science, policy and public opinion often remains complex, not only during exceptional circumstances, such as public health emergencies. Independence, scientific excellence, transparency and timeliness are essential qualities of credible recommendations. Beyond these attributes, effective communication is key, and SAGE aspires to make its decisions comprehensible and openly acknowledge data gaps, limitations and uncertainties. This is of particular importance during disease outbreaks where massive amounts of information (i.e., so-called infodemics), including false or misleading information by various media or individual sources, often overwhelms even knowledgeable and well-informed individuals.

Inevitably, SAGE needs to take into account novel ways of communication in a rapidly changing environment. As already remarked in the 2018 evaluation of SAGE [14], the advisory group could further improve the dissemination of its outputs. A lot of work has been performed since 2018 to facilitate access to information related to SAGE’s work. A recent effort has been the development of the search tool on the WHO website, which enables the user to scan SAGE-related meetings and documents for specific search terms [47]. That said, SAGE needs to remain vigilant to the rapid developments in the field of communication while ensuring that any new strategy is tailored to the needs of its target audience.

Special emphasis should be placed on maintaining SAGE’s strong linkage and relationship with RITAGs and NITAGs as well as with key actors in the immunization community, including international and non-governmental organizations, donors, manufacturers and other partners.

In the context of declining and competing financial and human resources, the capacity of countries to introduce novel vaccines into their immunization programs is increasingly saturated. The prioritization of vaccines and other public health measures in the current resource and funding environment, including dedicated cost-effectiveness analysis, is inherent to national programs and needs to be tackled at the country level. WHO and other stakeholders will continue to assist Member States with their vaccine and immunization prioritization exercises; efforts to develop respective tools are ongoing and will be reviewed by SAGE in 2025.

The ongoing mpox PHEIC has again reminded the global health community that crises necessitating a vaccination response will continue to occur, likely even at a higher frequency due to climate-change-related phenomena such as flooding, population displacements, encroachment on animal habitats, overcrowding and armed conflict. In this context, rapid, evidence-based guidance remains highly relevant and important. One of the IA2030 strategic priorities is to improve peoples’ and health authorities’ valuing of immunization. To reach this goal, IA2030 calls to strengthen evidence-based decision-making and points out the critical role of NITAGs. Stronger efforts are required to ensure that NITAGs and national immunization programs in low- and middle-income settings are adequately supported to take SAGE’s guidance and tailor context-specific vaccination recommendations.

WHO and its partners are working to strengthen country competencies and build regional hubs and centers of excellence to that effect. Notably, the Global NITAG Network (GNN) [48] is a platform for fostering collaborations as well as sharing experiences and best practices among NITAGs. The NITAG Resource Center [49] is a unique source of information, providing national immunization programs and NITAGs with evidence, information and training to develop national immunization policies on vaccine-preventable diseases. A more systematic approach has been set up to engage NITAGs with the SAGE plenary meetings and to disseminate the outcomes and formal recommendations to the NITAG community. Through the Global NITAG Network [17], regular webinars are conducted with NITAGs across the world to debrief the community on SAGE deliberations.

Technological innovations also have a role to play: artificial intelligence (AI)-aided approaches are facilitating the work in many fields, including public health. AI will have a profound impact on how evidence-based recommendations are developed. Scientific advisory bodies, including SAGE, need to anticipate and leverage these changes. Keeping abreast of these evolutions is paramount given that resource-constrained settings, which SAGE caters specifically to, may particularly benefit from these developments. Processes are evolving at this moment and have started to provide faster and resource-sparing solutions, including AI-performed evidence syntheses. Questions remain on their validity and policy-relevant interpretation. One future scenario may be for SAGE to provide policy options that can easily be tailored to the specific context with the help of AI.

In conclusion, over the last quarter century, SAGE has significantly contributed to shaping the vaccine and immunization landscape globally. It has earned and maintained a high level of integrity and credibility. The advisory group continues to be an authority in global public health, and its recommendations have profound implications for the health of individuals and populations across the globe. SAGE’s reach and impact depend on its relevance to regions and countries and for stakeholders and partners to build on SAGE’s recommendations. The contextualization of recommendations to country needs, priorities, and capacities has gained major importance, and SAGE will assess strategies and tools to assist countries in prioritizing vaccines at the national level.

Against the backdrop of the plethora of crises and emerging global challenges, such as climate change, migration, armed conflict, humanitarian emergencies and aging populations, continuous critical revision and adaptation of SAGE’s scope and working processes is key to ensuring the appropriateness of its guidance and relevance to the WHO Member States and partners in the decades to come.

## Figures and Tables

**Table 1:** New or updated vaccine position papers by year.

**YEAR**	**1998**	**1999**	**2000**	**2001**	**2002**	**2003**	**2004**	**2005 ^**	**2006**
**Vaccine position paper**	Varicella vaccines * Japanese encephalitis vaccines *	Rotavirus vaccines * Pertussis vaccines *	**Rubella vaccines**	Cholera vaccines * Mumps virus vaccines *	Rabies vaccines * Meningococcal vaccines: polysaccharide and poly-saccharide conjugate vaccines *	Rotavirus vaccines Pneumococcal vaccines * Yellow fever vaccine *	BCG Vaccine * Measles vaccines * Hepatitis B vaccines *	None	Diphtheria vaccines * Tetanus vaccine * Japanese Encephalitis Vaccines Haemophilus *influenzae* type b (Hib) conjugate vaccines *
**YEAR**	**2007**	**2008**	**2009**	**2010**	**2011**	**2012**	**2013**	**2014**	**2015**
**Vaccine position paper**	**Mumps virus vaccines** * Rabies vaccine Pneumococcal conjugate vaccine Rotavirus vaccines Rabies vaccines	**Typhoid vaccines** * 23-valent pneumococcal poly- saccharide vaccine	**Human papilloma-** **virus vaccines** * Measles vaccines Hepatitis B vaccines Rotavirus vaccines	Cholera vaccines **Polio vaccines and polio immunization in the pre-eradication era** * Rabies vaccines	Vaccines against tick-borne encephalitis* Rubella vaccines * Meningococcal vaccines	Pneumococcal vaccines * Hepatitis A vaccines * Vaccines against influenza *	Rotavirus vaccines Vaccines and vaccination against yellow fever HiB vaccination	Polio vaccines **Varicella and herpes zoster vaccines** * Human papilloma-virus vaccines	Japanese Encephalitis Vaccines Meningococcal A conjugate vaccine **Hepatitis E vaccines** Pertussis vaccines Reducing pain at the time of vaccination *
**YEAR**	**2016**	**2017**	**2018**	**2019**	**2020**	**2021**	**2022**	**2023**	**2024**
**Vaccine position paper**	Malaria vaccine * Polio vaccines Dengue vaccine *	Tetanus vaccines Measles vaccines Human papilloma-virus vaccines Hepatitis B vaccines Diphtheria vaccines Cholera vaccines	BCG vaccines Typhoid vaccines Rabies vaccines Dengue vaccines	Pneumococcal conjugate vaccines in infants and children under 5 years of age	Rubella vaccines	Pneumococcal vaccines/their use in community outbreak settings Rotavirus vaccines	Malaria vaccine Vaccines against influenza **Understanding the behavioral and social drivers of vaccine uptake** Polio vaccines Hepatitis A vaccines Human papillomavirus vaccines	None	Meningococcal vaccines/use of multivalent meningococcal conjugate vaccines in countries of the African meningitis belt Mumps virus vaccines Dengue vaccines Malaria vaccine Smallpox and mpox (orthopoxviruses) vaccine *

^ SAGE took on a formal role in revising the WHO vaccine position papers as of 2005. * First position paper on a specific topic.

## Data Availability

Data sharing is not applicable.
